# Polyclonal Antibodies Derived from Transchromosomic Bovines Vaccinated with the Recombinant F1-V Vaccine Increase Bacterial Opsonization In Vitro and Protect Mice from Pneumonic Plague

**DOI:** 10.3390/antib12020033

**Published:** 2023-05-08

**Authors:** Sergei S. Biryukov, Hua Wu, Jennifer L. Dankmeyer, Nathaniel O. Rill, Christopher P. Klimko, Kristi A. Egland, Jennifer L. Shoe, Melissa Hunter, David P. Fetterer, Ju Qiu, Michael L. Davies, Christoph L. Bausch, Eddie J. Sullivan, Thomas Luke, Christopher K. Cote

**Affiliations:** 1 Bacteriology Division, United States Army Medical Research Institute of Infectious Diseases, 1425 Porter Street, Fort Detrick, Frederick, MD 21702, USA; 2 SAB Biotherapeutics, 2100 E 54th St. N, Sioux Falls, SD 57104, USA; 3 Biostatistics Division, United States Army Medical Research Institute of Infectious Diseases, 1425 Porter Street, Fort Detrick, Frederick, MD 21702, USA

**Keywords:** *Yersinia pestis*, antibodies, transchromosomic bovine, plague, mice, opsonization, recombinant F1-V vaccine

## Abstract

Plague is an ancient disease that continues to be of concern to both the public health and biodefense research communities. Pneumonic plague is caused by hematogenous spread of *Yersinia pestis* bacteria from a ruptured bubo to the lungs or by directly inhaling aerosolized bacteria. The fatality rate associated with pneumonic plague is significant unless effective antibiotic therapy is initiated soon after an early and accurate diagnosis is made. As with all bacterial pathogens, drug resistance is a primary concern when developing strategies to combat these *Yersinia pestis* infections in the future. While there has been significant progress in vaccine development, no FDA-approved vaccine strategy exists; thus, other medical countermeasures are needed. Antibody treatment has been shown to be effective in animal models of plague. We produced fully human polyclonal antibodies in transchromosomic bovines vaccinated with the recombinant F1-V plague vaccine. The resulting human antibodies opsonized *Y. pestis* bacteria in the presence of RAW264.7 cells and afforded significant protection to BALB/c mice after exposure to aerosolized *Y. pestis*. These data demonstrate the utility of this technology to produce large quantities of non-immunogenic anti-plague human antibodies to prevent or possibly treat pneumonic plague in human.

## 1. Introduction

*Yersinia pestis* is a gram-negative Tier 1 select bacterial biothreat agent that can cause rapidly fatal infections [1,2,3]. While bubonic plague is the most common form of the disease, pneumonic plague is the primary concern in the context of biodefense scenarios [4,5]. *Y. pestis* is a major biothreat due to its capacity for aerosol dissemination and its contagious nature in the pneumonic form. The illness can be treated with several different classes of antibiotics, including aminoglycosides (e.g., streptomycin) and quinolones (e.g., ciprofloxacin) [6,7]. However, antibiotic treatment options could become limited if the bacteria acquire antibiotic resistance either through natural means or if engineered by an adversary [8,9]. Recent outbreak events in Madagascar and documented examples of naturally acquired antibiotic resistance emphasize the need for novel therapeutics that can be used either alone or in combination [10,11,12,13].

Two protective antigens have been used to make subunit vaccines, including the F1 capsular antigen and the LcrV antigen [14,15]. The F1 protein is encoded by the *caf1* gene located on a large plasmid (pMT) and is robustly expressed at 37 °C [16,17]. F1 inhibits the uptake of the bacteria by macrophages by creating an anti-phagocytic capsule [18,19,20]. It is also thought to play a role in bacterial transmission because it inhibits the adhesion of the bacteria to human epithelial cells [21]. However, strains of *Y. pestis* that are F1 negative (e.g., C12 strain) have been identified and retain their virulence in mice [22,23,24,25,26], thereby emphasizing the need for combination vaccine strategies, including other protective antigens such as the LcrV protein [27,28]. The LcrV antigen is encoded with other type-3 secretion system (T3SS) proteins on pCD1 and is a major virulence factor that localizes to the tip of the T3SS [29,30]. This antigen facilitates Yop translocation, which results in the inhibition of phagocytosis, induction of apoptosis, and *Yersinia*-induced immune suppression [31,32,33,34]. The LcrV antigen has also been demonstrated to be a multifactorial protein as it can be translocated into host cells and plays various roles in bacterial pathogenesis [31,35,36]. The protective epitope of the LcrV antigen has been mapped by several groups and includes amino acids 135 to 275 [31,37]. Active immunization with recombinant LcrV protein was previously shown to confer protection against both the bubonic and pneumonic models of plague caused by both the encapsulated CO92 strain and the F1 negative strain C12 [28]. However, the level of protection against non-encapsulated strains remains equivocal. The combination of both F1 and LcrV vaccine antigens resulted in improved protection in mice infected with *Y. pestis*. The dual antigens were theorized to be able to protect against emerging/engineered threats that may be F1 negative in spite of known heterogeneity amongst the LcrV proteins from different isolates [38,39,40]. Researchers in the United States have pursued a chimeric protein strategy (i.e., rF1-V), whereas researchers in the United Kingdom focused their efforts on a vaccine with both distinct protein entities (i.e., F1 + V) [41,42,43,44,45,46]. However, to date, there is no FDA-approved vaccine to prevent or ameliorate plague.

The vaccine studies using the F1 and LcrV antigens suggest that antibodies play a role in protection. When administered prophylactically or 48 h post-infection, either alone or in combination, passive immunization with two monoclonal antibodies (mAbs) generated against *Y. pestis* LcrV (mAb 7.3) and F1 (mAb F1-04-A-G1) antigen is protective in mouse models of bubonic and pneumonic plague [47]. The in vivo protection afforded by the anti-LcrV antibody has been shown to correlate in vitro with increased phagocytosis by macrophages and reduced macrophage cell death following infection with *Y. pestis* [31].

Passive protection has been accomplished using antibodies directed against either the F1 or the LcrV antigen [37,44,48,49,50,51,52,53,54]. Both mouse- and human-derived anti-F1 mAbs have been demonstrated to protect mice against *Y. pestis* infection [48,49,55]. The anti-LcrV mAb 7.3 antibody is an IgG1 and also protects against plague disease [37,47,56]. Both anti-F1 and anti-LcrV antibodies increased phagocytic uptake of *Y. pestis* by macrophages and protected macrophages from *Y. pestis*-induced cell death. The observed in vitro phenotype, however, is dependent upon the culture conditions used to grow the bacteria in the laboratory, as these conditions affect the expression of these protective antigens by the bacteria.

In this report, we characterize and evaluate anti-plague polyclonal antibodies generated in transchromosomic (Tc) bovines using ELISA, functional macrophage assays, and in vivo mouse models of pneumonic plague. Tc bovines endogenously produce fully human IgG polyclonal antibodies in response to environmental and vaccine-delivered antigens [57,58,59]. When hyperimmunized with one or more antigens, very high titers can be achieved in their plasma, and the IgG antibodies can be highly purified into a specific immunoglobulin with mainly IgG1 subtype. Experimental therapeutics derived from Tc bovines have shown excellent efficacy in preclinical studies to other select agents and virulent pathogens including Ebola virus, Venezuelan equine encephalitis virus, severe acute respiratory syndrome coronavirus 2 (SARS-CoV-2), and Middle East respiratory syndrome coronavirus (MERS-CoV), among others [57,58,59,60,61,62]. This approach could potentially be used to develop a polyclonal plague countermeasure using F1, LcrV, and/or other antigens in combination.

## 2. Materials and Methods

### 2.1. Bacterial Strains and Growth Conditions

The *Y. pestis pgm*- pPst- strain was generated at USAMRIID and was kindly provided by Susan Welkos (USAMRIID, Frederick, MD, USA) [49]. *Y. pestis pgm*- pPst- is an attenuated strain derived from the fully virulent *Y. pestis* CO92 strain, which is cured of the pPst plasmid containing the plasminogen activator (Pla) virulence locus (*pla*) and is pigmentation locus (*pgm*)-deficient [63,64]. *Y. pestis* was grown on Remel^®^ Sheep Blood Agar (SBA) plates (Thermo Fisher Scientific, Waltham, MA, USA) and incubated at 28 °C or 37 °C for 24 h. Bacterial colonies were harvested and used to inoculate 10 mL of brain heart infusion (BHI) broth (BD Biosciences, San Jose, CA, USA) and incubated in the BHI medium for 2 h at 37 °C with shaking at 200 rpm prior to infecting the macrophages. To ensure the bacteria are harvested in the log phase of growth, the OD_600_ of the culture post-incubation was not allowed to exceed 1.0 prior to incubation with antibodies. For in vivo challenge studies the fully virulent *Y. pestis* CO92 was used. Broth cultures were inoculated using growth from freshly inoculated tryptose blood agar base (BD Biosciences, San Jose, CA, USA) slants (grown at 28–30 °C for approximately 48 h) which were suspended in Heart Infusion broth (BD Biosciences, San Jose, CA, USA) + 0.2% Xylose (Sigma Aldrich, St. Louis, MO, USA) (HIBX) and incubated approximately 24 h at 28–30 °C and shaking at 150 rpm.

### 2.2. Mouse Monoclonal Antibodies

The anti-F1 mouse IgG1 monoclonal antibody (mAb) F1-04-A-G1 was provided by James Burans and Jennifer Aldrich (Naval Medical Research Center, Silver Spring, MD, USA) [49]. The anti-LcrV mouse IgG1 mAb (7.3) was provided by Jim Hill (DSTL Porton Down, Salisbury, UK) and has been described previously [31,37,47,49,56].

### 2.3. Production of Anti-rF1-V Human Polyclonal Antibodies SAB-183 from Transchromosomic (Tc) Bovines

Tc bovines were produced as previously described [65,66]. The Tc bovines used in this study are homozygous for triple deletion in the endogenous bovine immunoglobulin genes (*bIGHM* ^− / −^, *bIGHML1* ^− / −^, *bIGL* ^− / −^) and carry a human artificial chromosome (HAC) vector labeled as isKcHACD with an IgG1 production bias. This HAC vector consists of human chromosome 14 fragment and 2 fragment. The 14 fragment contains the entire human immunoglobulin heavy chain locus except that the IGHM constant region remains bovine, and the key regulatory sequences were bovinized. The 2 fragment contains the entire human immunoglobulin k light chain locus [65,66].

### 2.4. Tc Bovine Immunization and Plasma Collection

Two Tc bovines were immunized with 2 mg recombinant F1-V (rF1-V), a fusion protein of the F1 capsular antigen and the virulence-associated LcrV gene product, formulated with SAB’s proprietary adjuvant formulation (SAB-adj-1) for the first vaccination (V1) and the second vaccination (V2) at a 3-week interval. The bovines were then boosted with 5 mg rF1-V formulated with SAB-adj-1 for third vaccination (V3) to seventh vaccination (V7) at 4-week intervals. Recombinant F1-V, produced with fermentation and expressed in *E. coli* and purified, was provided by DynPort Vaccine Company (Frederick, MD, USA) through the Joint Program Executive Office (JPEO) for Chemical and Biological Defense. Up to 2.1% of body weight of hyperimmune plasma per animal was collected from immunized Tc bovines on days 8, 11, and 14 after vaccination V3 throughV7. Plasma was collected using an Autopheresis C, Model 200, automated plasmapheresis system (Baxter Healthcare, Deerfield, IL, USA). Plasma samples were stored frozen at −20 °C until purifications were performed.

### 2.5. cGMP Purification of SAB-183

SAB-183 (lot PD2001332PG) was purified from pooled Tc bovine plasma from V3 to V7 as previously described [67]. Negative control antibody preparation (PR1701041NC) was purified from Tc bovine pre-immune plasma.

### 2.6. Cell Culture

RAW264.7 murine macrophage-like cells derived from an Abelson murine leukemia virus tumor (ATCC TIB-71) were grown at 37 °C in 5% CO_2_ in low glucose Dulbecco’s Modified Eagle Medium (DMEM) containing (Corning, Manassas, VA, USA) 1% L-glutamine, 1% non-essential amino acids, 1% HEPES buffer (Sigma-Aldrich, St. Louis, MO, USA), and 10% fetal bovine serum (Hyclone, Thermo Fisher Scientific, Waltham, MA, USA). Cells were used before passage 15 and seeded in 96-well plates using an automated Multidrop Combi Reagent Dispenser (Thermo Fisher Scientific, Waltham, MA, USA).

### 2.7. Quantification of Viable Intracellular Y. pestis (Gentamicin Protection Assay)

**Figure 1:**
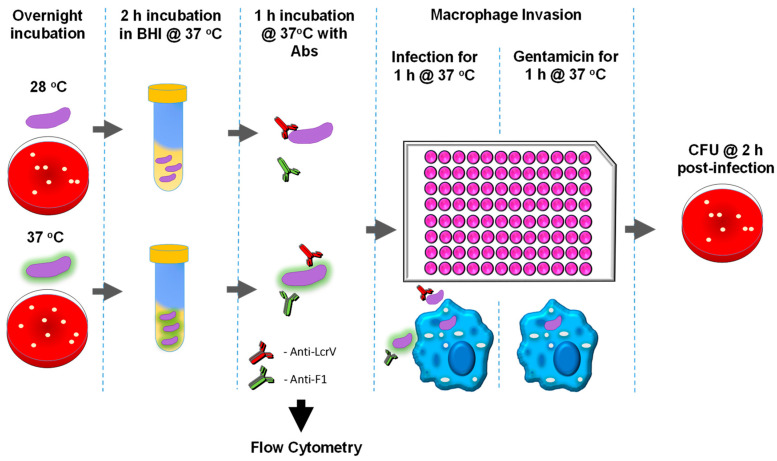
Schematic representation of gentamicin protection assay using *Y. pestis* grown at different temperatures to enrich for specific antigens.

A schematic representation of this assay is provided in Figure 1. Bacterial cultures were suspended in DMEM from cultures grown in BHI broth, and multiplicity of infection (MOI) was estimated for an OD_600_ of 1.0 (~5.34 × 10^8^ colony forming units (CFU) per milliliter). Depending upon the targeted protein, the in vitro assays were performed using bacteria grown under different temperatures to adequately characterize the antibody–bacteria interactions. *Y. pestis* requires at least 4 h of growth at 37 °C prior to infection to produce enough capsule to prevent phagocytosis [18], while an hour or less at 37 °C is sufficient to observe T3SS-inhibited phagocytosis [68,69]. For macrophage infection assays, cells (1.5 × 10^4^ cell/well) were seeded into 96-well plates one day prior to infection. *Y. pestis*, at 8 × 10^6^ CFU/mL was pre-incubated with 10 µg/mL or 100 µg/mL antibodies in DMEM for 1 h at 37 °C prior to infection. Macrophages were then infected at an MOI of approximately 10, in triplicate wells. The plates were centrifuged at 200× *g* for 5 min to initiate infection and then incubated at 37 °C with 5% CO_2_. After 1 h of infection, gentamicin (8 μg/mL) (Sigma-Aldrich, St. Louis, MO, USA) was added to the wells to kill extracellular bacteria, and the plates were incubated for an additional hour at 37 °C with 5% CO_2_. After incubation macrophages were washed two times in PBS and lysed using 0.1% Triton X-100 in PBS. Serial dilutions of lysates were plated in duplicate on SBA plates and incubated for 2 days at 28 °C for CFU enumeration. Additional control wells were also infected with *Y. pestis* that was not pre-incubated with any antibody (Yp only).

### 2.8. Exposure of Mice to Aerosolized Y. pestis

Aerosolized challenge doses of virulent *Y. pestis* CO92 (pneumonic plague model) were prepared as previously described [70,71]. The cultures were harvested with centrifugation and suspended in HIB medium (no xylose) to the estimated concentration yielding the desired number of LD_50_ equivalents. Exposure of mice to aerosolized bacteria was accomplished as previously described [70,71]. Briefly, 7- to 9-week-old female BALB/c mice (Charles River, Frederick, MD, USA) were transferred to wire mesh cages and placed in a whole-body aerosol chamber within a Class III biological safety cabinet located inside a BSL-3 laboratory. Mice were exposed to aerosolized *Y. pestis* strain CO92 (encapsulated) created with a three-jet collision nebulizer. Samples were collected from the all-glass impinger (AGI) vessel and analyzed with CFU calculations to determine the inhaled dose of *Y. pestis.* The median lethal dose for *Y. pestis* CO92 in female BALB/c mice is approximately 6.8 × 10^4^ inhaled CFUs [26,72].

### 2.9. ELISA

Immunoglobulin (Ig) class IgG titers (IgG, IgG1, IgG2a) from vaccinated bovines were determined with an ELISA performed in 96-well, Immulon 2 HB, round-bottom plates (Thermo Fisher Scientific, Waltham, MA, USA). Recombinant F1-V (cGMP; DynPort Vaccine Company, Frederick, MD, USA), F1 (BEI Resources, Manassas, VA, USA), and LcrV (BEI Resources. Manassas, VA, USA) were individually used as antigens diluted in 0.1 M carbonate buffer, pH 9.5, to a concentration of 2 µg/mL. Irradiated temperature-shifted *Y. pestis* CO92 (TS CO92) and its non-encapsulated derivative strain *Y. pestis* C12 (TS C12) were diluted, as described above, but at a concentration of 10 µg/mL [24,64]. Plates were covered and stored overnight at 4 °C. The plates were washed five times with wash buffer (PBS, 0.05% Tween 20) with a Biotek ELx405ts plate washer (Bio Tek, Winooski, VT, USA), and incubated with blocking buffer (1% Casein in PBS, Thermo Fisher Scientific, Waltham, MA, USA) for 30 min at 37 °C. Blocking buffer was removed with washing as stated above, then twofold serial dilutions of bovine sera were made with antibody assay diluent (BS, 0.25% Casein) in triplicate, and plates were incubated for 1 h at 37 °C. Then the plates were washed as previously mentioned; diluted anti-IgG horseradish peroxidase conjugate at 1:5,000 (Southern Biotechnology Associates, Inc., Birmingham, AL, USA) was added to each well and plates were incubated for 30 min at 37 °C. After the plates were washed as previously stated, buffered hydrogen peroxide and 3,3′,5,5′-tetramethylbenzidine solution (Thermo Fisher Scientific, Waltham, MA, USA) was added to each well, and plates were incubated for 20 min at 37 °C. The reaction was stopped with 2 N sulfuric acid, and the amount of bound antibody was determined colorimetrically with readings at 450 nm with a reference filter (570 nm) using a Biotek ELx808 plate reader (Bio Tek, Winooski, VT, USA). The results are reported as the reciprocal of the highest dilution giving a mean OD of at least 0.1 (which was at least twice the background) ±1 SD.

### 2.10. Flow Cytometry

Approximately 4 × 10^5^ *Y. pestis* CO92 *pgm*-/pPst- (Yp *pgm*-/pPst-) cells were treated with anti-F1 or anti-LcrV antibodies as described above. The inoculum samples were centrifuged at 2800× *g* for 10 min and resuspended in 350 µL dPBS (Gibco, Thermo Fisher Scientific, Waltham, MA, USA). The samples were then read on a FACSCanto II flow cytometer (BD Biosciences, San Jose, CA, USA), gating on *Y. pestis pgm*-/pPst- untreated cells in the FSC vs. SSC dot plot and noting the aggregation and shift of subsequent antibody-treated *Y. pestis pgm*-/pPst- bacterial cells outside of that gate.

For assessment of F1 and LcrV levels on bacterial surface, Yp *pgm*-/pPst- cells were resuspended in 1X dPBS, then diluted to 8 × 10^6^ CFU/mL in DMEM and pre-incubated with 10 µg/mL primary antibody (F1-04-A-G1 or 7.3) for 1 h at 37 °C. After washing in FACS buffer (DPBS + 1% bovine serum albumin [HyClone]), cells were resuspended in FACS buffer with secondary goat anti-mouse antibody (Alexa Fluor 488 conjugate, Invitrogen) at 10 µg/mL. After 30 min at room temperature, cells were washed, resuspended in FACS buffer, and read on a FACSCanto II.

### 2.11. Statistics

For ELISA, an exact, one-sided, two-sample Wilcoxon test was performed between treatments. No adjustment was applied for multiple comparisons. The comparison was made between the three technical replicates taken on each animal. For in vitro macrophage assays and gentamicin protection assay, results were compared using a Wilcoxon rank sum test, stratified by date of experiments. The survival rates at selected time points were compared using Fisher exact test. The log-rank test was used to compare mouse survival curves post challenge. The ED_50_ is estimated at selected time points with logistic regression. In addition, a predicted value at each day postexposure is given with accelerated failure time model. Any *p* values of ≤ 05 were considered significant. Analyses were performed in SAS version 9.4 (SAS Institute Inc., Cary, NC, USA).

## 3. Results

### 3.1. Mouse Anti-F1 and Anti-LcrV mAbs and Polyclonal Anti-rF1-V Antibodies Derived from Transchromosomic (Tc) Bovines Are Opsonic In Vitro

We initially compared the human polyclonal anti-rF1-V antibodies (SAB-183), produced in Tc bovines immunized with the recombinant F1-V fusion protein vaccine, to the well-characterized mouse monoclonal (mAbs) anti-LcrV (7.3) and anti-F1 (F1-04-A-G1), which have previously been shown to be highly protective in mice against *Y. pestis* challenge. Furthermore, in an effort to discern how the human polyclonal antibodies affect phagocytosis and/or growth of *Y. pestis pgm*-/pPst- within host macrophages, we first optimized the *Y. pestis* growth conditions to induce expression of selective temperature-dependent virulence factors. For instance, at low or ambient temperature (26–28 °C), similar to that in the flea vector, there is little to no expression of the F1 capsule protein [18,19,20]. Subsequent transition to 37 °C, a temperature that mimics a mammalian host, results in the induction of F1 expression. It has been previously shown that *Y. pestis* needs to be grown at 37 °C for >2 h before the anti-phagocytic activity of the capsule is appreciable [18,31]. In order to capitalize on this temperature-dependent bacterial growth characteristic, *Y. pestis* was initially grown at 28 °C or 37 °C for 24 h on SBA plates and then was sub-cultured in BHI medium and grown for an additional 2 h at 37 °C. *Y. pestis* grown at 28 °C for 24 h followed by 37 °C for 2 h (28–37 °C) expresses a very limited F1 capsule that would not likely obscure the LcrV antigen, a component of the T3SS, on the surface of the bacterial cell. In contrast, *Y. pestis* grown at 37 °C for 24 h followed by 37 °C for 2 h (37–37 °C) expresses a much more robust F1 capsule and also appreciable levels of the LcrV antigen. These bacterial cell descriptions based upon the previous literature were confirmed in our laboratory with Western blot and flow cytometric analyses (Supplementary Figure S1).

**Figure 2:**
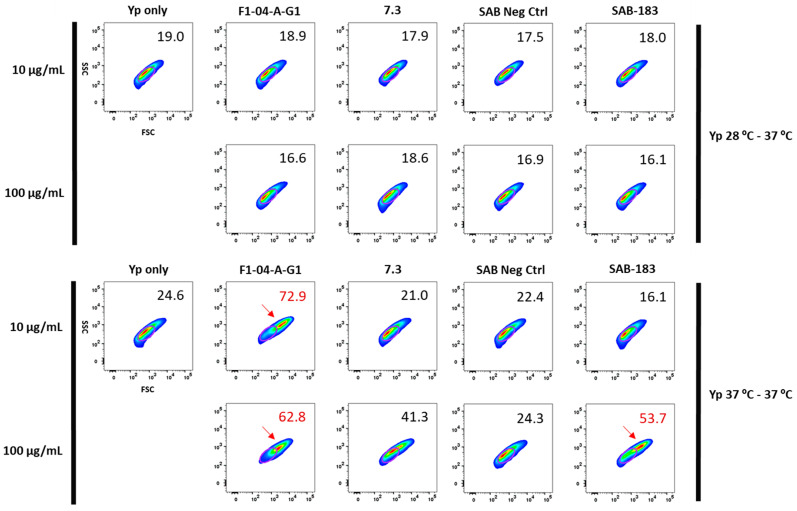
The mouse and human anti-F1 antibodies promote bacterial aggregation. *Y. pestis* CO92 *pgm*- pPst- (**Top**) 28–37 °C and (**Bottom**) 37–37 °C was incubated for 1 h with 10 μg/mL or 100 μg/mL of antibodies prior to analyzing the bacteria populations with gating, using FSC (size, x-axis) and SSC (granularity, y-axis). The numbers denote the percentage of the bacterial cells outside of the gate. Red arrow demarcates bacterial population size and granularity shift relative to no Ab (Yp only) pre-incubation. Shown here are data from one representative experiment from a total of three independent experiments, all with similar results.

In order to evaluate if anti-F1, anti-LcrV, or polyclonal anti-rF1-V antibodies affected bacteria post-incubation we incubated *Y. pestis* grown at 28–37 °C or 37–37 °C with mouse monoclonal anti-F1 (F1-04-A-G1), anti-LcrV (7.3) mAbs, or with human polyclonal anti-rF1-V antibodies (SAB-183) derived from Tc bovines. Changes in the bacterial population, such as alterations in size or granularity, were assessed with flow cytometry. Incubation of *Y. pestis* grown at 28–37 °C with antibodies resulted in no overt change in bacteria relative to *Y. pestis* cells in the absence of antibodies (Yp only) or human polyclonal antibody from non-immunized Tc bovines (SAB Neg Ctrl) (Figure 2-Top). After incubation of *Y. pestis* grown at 37–37 °C with 10 µg/mL or 100 µg/mL of F1-04-A-G1 mAb, there was a noticeable change in size and granularity of the bacterial population. Further supporting the lack of availability of the LcrV protein to interact with antibodies (compared to the F1 protein), the bacteria grown at 37 °C −37 °C and incubated with 100 μg/mL of anti-LcrV antibody 7.3 exhibited an appreciable alteration as detected using flow cytometry, but the degree of aggregation was clearly less than that observed when the bacteria were incubated with the anti-F1 mAb, and this observation is not consistent between iterations. Although no change in bacteria was observed in the presence of 10 µg/mL of polyclonal SAB-183 antibodies, there was a marked shift in the bacterial population in the presence of 100 µg/mL of polyclonal SAB-183 material (Figure 2-Bottom).

**Figure 3:**
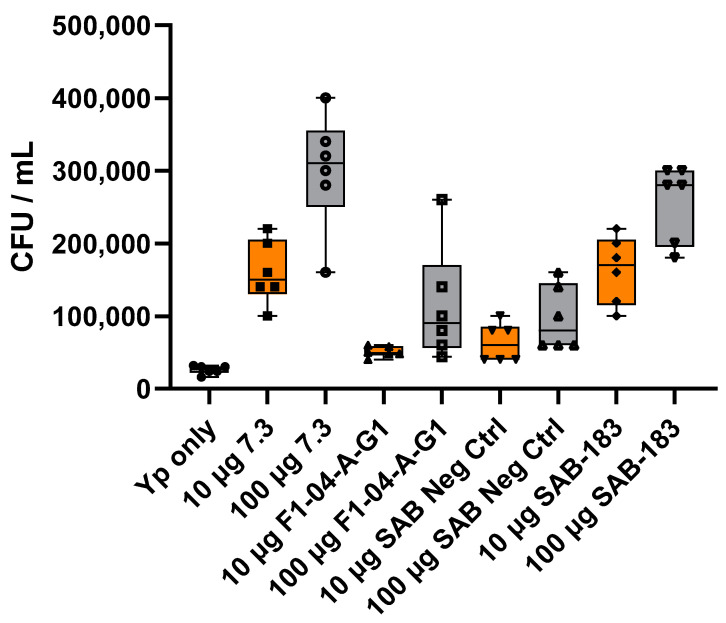
The mouse and human anti-LcrV antibodies are opsonic in vitro. *Y. pestis* CO92 *pgm*- pPst- (28–37 °C) was incubated for 1 h with 10 μg/mL (orange) or 100 μg/mL (grey) of antibodies prior to infection of RAW264.7 cells at an MOI of approximately 10 CFU. Two hours post-infection, macrophages were lysed plated in duplicate on SBA plates for CFU enumeration. The box-plots depict the median value, each technical replicate in that iteration, and the 1st and 3rd quartile values. This is a representative experiment of five similar experiments. Statistical analyses of two iterations are provided in Supplementary Table S1.

Mouse anti-LcrV mAb (7.3) enhanced initial opsonization and phagocytosis of *Y. pestis* 28–37 °C at 2 h post-infection. The level of bacterial internalization was more pronounced with *Y. pestis* pre-incubated with 100 μg/mL of mAb compared to *Y. pestis* preincubated with 10 μg/mL. The levels of phagocytosis enhancement were also similarly observed with SAB-183 polyclonal antibodies relative to the anti-LcrV mAb (7.3) (Figure 3).

**Figure 4:**
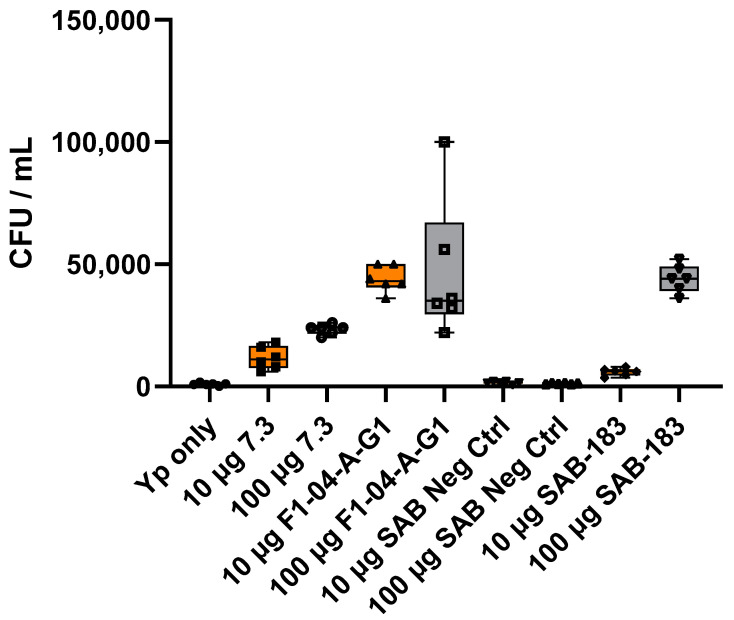
The mouse and human anti-F1 antibodies are opsonic in vitro. *Y. pestis* CO92 *pgm*- pPst- (37–37 °C) was incubated for 1 h with 10 μg/mL (orange) or 100 μg/mL (grey) of antibodies prior to infection of RAW264.7 cells at an MOI of approximately 10 CFU. Two hours post-infection, macrophages were lysed plated in duplicate on SBA plates for CFU enumeration. The box-plots depict the median value, each technical replicate in that iteration, and the 1st and 3rd quartile values. This is a representative experiment of five similar experiments. Statistical analyses of two iterations are provided in Supplementary Table S1.

Mouse anti-F1 mAb (F1-04-A-G1) opsonized and enhanced phagocytosis of *Y. pestis* 37–37 °C at 2 h post-infection. Both capsule production and T3SS upregulation are induced at 37 °C, therefore enhancement of bacterial uptake is observed with both anti-F1 (F1-04-A-G1) and anti-LcrV (7.3). However, the increase in phagocytosis is substantially greater in the presence of the anti-F1 mAb relative to the anti-LcrV mAb, likely due to increased F1 production and overall epitope availability. The levels of phagocytosis enhancement also reached statistical significance post-incubation with SAB-183 relative to the *Y. pestis* only or the SAB Negative Control. (Figure 4).

### 3.2. Transchromosomic (Tc) Bovines Immunized with rF1-V Elicit a Strong Human Antibody Response

Previous studies identified that alterations in vaccine formulations, such as inclusion of various adjuvants, aside from impacting antibody titers can also change the epitope binding profile of the polyclonal antibody responses, with some epitopes conferring superior protection versus others on the same antigen [73,74]. In an effort to discern how the bovine rF1-V vaccination formulation and immunization schedule impact antibody response, we measured total antibody response by means of indirect ELISA against F1- and LcrV-protein antigens, along with irradiated whole-cell *Y. pestis* temperature-shifted CO92 (TS CO92) and irradiated whole-cell *Y. pestis* temperature-shifted non-encapsulated C12 (TS C12) strains.

During antigen preparation both strains of *Y. pestis* were grown at 28 °C and then temperature switched (TS) to 37 °C for approximately 3.5 h in order to upregulate the expression of the bacterial capsule and T3SS components. The anti-F1 and anti-LcrV antibody responses were significantly increased in the SAB-183 material relative to the SAB Negative Control (*p* < 0.05 vs SAB Negative Control). The antibody response was substantially greater against the LcrV antigen (8.3 × 10^4^–2.4 × 10^5^) relative to the F1 antigen (1.6 × 10^4^–2.4 × 10^4^) (Table 1). In addition, a significant antibody response (*p* = 0.05 vs SAB Negative Control) was also elicited against *Y. pestis* TS CO92 and *Y. pestis* TS C12. The antibody response was higher against *Y. pestis* TS CO92 (4.2 × 10^3^–8.8 × 10^3^) relative to *Y. pestis* TS C12 (1.3 × 10^3^–1.4 × 10^3^) (Table 1), likely due to the presence of the immunodominant F1 protein on the surface of the irradiated TS CO92 cells used as capture antigen.

### 3.3. Human Anti-rF1-V Antibodies Derived from Transchromosomic (Tc) Bovines can Protect Mice after Exposure to Aerosolized Y. pestis

**Figure 5:**
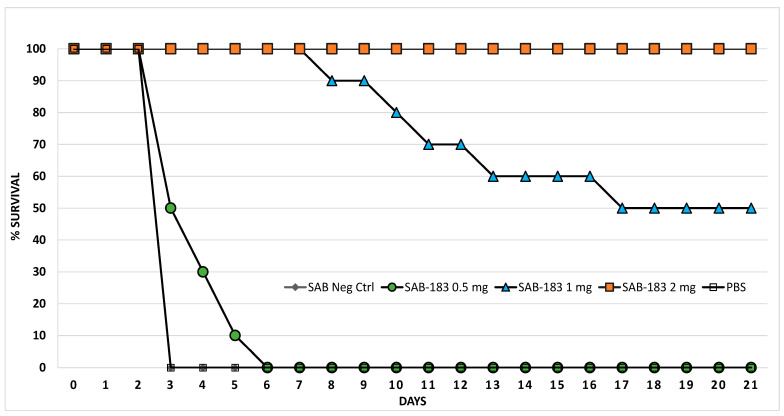
Anti-rF1-V antibodies derived from Tc bovines can protect mice from pneumonic plague. Mice were treated with 2.0 mg, 1.0 mg, or 0.5 mg of polyclonal IgG derived from Tc bovines vaccinated with the rF1-V plague vaccine (*n* = 10 for each group). Negative control animals (SAB Neg) were treated with 2.0 mg of polyclonal IgG derived from naïve Tc bovines *(n* = 7) or PBS (*n* = 4). There were no differences between the negative control groups, and the data coincide exactly on the graph. The positive control antibodies used were 0.2 mg anti-F1 (F1-04-A-G1) mouse-derived mAb (*n* = 3), or 0.1 mg anti-LcrV (7.3) mouse-derived mAb (*n* = 3). For clarity, the positive control antibodies are not depicted on the graph, but each protected 100% of the mice. All antibodies were delivered via intraperitoneal injection approximately 12 h prior to exposure at approximately 13 LD_50_ equivalents of aerosolized *Y. pestis* CO92.

Mice were treated with antibodies approximately 12 h pre-exposure to aerosolized *Y. pestis* CO92. Mice received 0.5 mg, 1.0 mg, or 2.0 mg of IgG purified antibodies derived from Tc bovines vaccinated with the rF1-V vaccine. Mice were then estimated to have inhaled approximately 8.6 × 10^5^ CFU of *Y. pestis* CO92 (approximately 13 LD_50_ equivalents). While all mice that received 0.5 mg of this polyclonal antibody succumbed to infection, there was a statistically significant delay in time-to-death or euthanasia compared to mice receiving PBS alone (*p* = 0.0003) or mice receiving purified IgG derived from Tc bovines not vaccinated with rF1-V (SAB Negative Control, *p* < 0.0001) (Figure 5). There was a significant dose-dependent response as survival correlated with the amount of anti-rF1-V polyclonal antibodies administered to the mice (See Figure 5). Day 21 survival rates were significantly greater when comparing the 0.5 mg treatment group with the 1.0 mg treatment group (*p* = 0.033) or with the 2.0 mg treatment group (*p* < 0.001). Likewise, there was a significant increase in survival rate when comparing the 1.0 mg treatment group with the 2.0 mg treatment group (*p* = 0.033).

For the animals that did succumb to infection or that were euthanized in accordance with early endpoint euthanasia criteria the time-to-death was similarly significant in a dose-dependent manner. When comparing the 0.5 mg treatment group with the 1.0 mg and 2.0 mg treatment groups, these differences in time-to-death were statistically significant (*p* < 0.001 in both comparisons and *p* = 0.012 when comparing 1.0 mg and 2.0 mg treatment groups) (Figure 5). Mouse-derived monoclonal antibodies were used as positive controls. Mice (*n* = 3) received either 0.1 mg of anti-LcrV mAb 7.3 or 0.2 mg of anti-F1 mAb F1-04-A-G1 and all positive control mice survived the infection.

**Figure 6:**
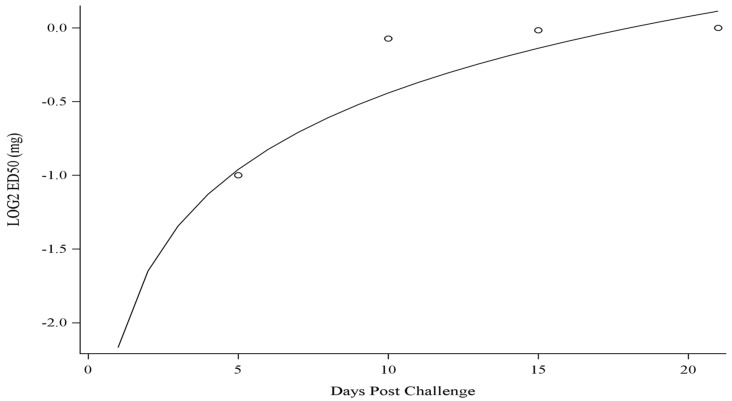
The ED_50_ is estimated from the mice (*n* = 30) treated with SAB-183 (0.5, 1, or 2 mg). The ED_50_ is estimated at selected time points using logistic regression (points in figure). In addition, a predicted value at each day postexposure is given using accelerated failure time model (solid line in figure).

The median effective dose (ED_50_) of the polyclonal material was calculated for days 5, 10, 15, and 21 postexposure to aerosolized *Y. pestis* (Figure 6). Through day 5 postexposure the ED_50_ is approximately 0.5 mg. For the remainder of the time points the ED_50_ was determined to be approximately 1.0 mg. The response to the treatment could change as the disease progresses or as the immune system mounts a response. The treatment is most effective early in the course of the disease, as indicated by the lower ED_50_ on day 5 compared to the higher ED_50_ on day 14 and 21. Overall, the variation in the ED_50_ over time suggests that the timing and dosing of the treatment may be important factors in achieving the desired prophylactic or therapeutic effect.

## 4. Discussion

Having the ability to screen for promising antibody candidates in vitro could reduce the number of animals that would have to be utilized in passive transfer studies, expedite the timeline of the screening process, reduce the initial quantity of the antibody required for initial evaluation, and significantly lower the overall discovery cost. This study encompassed two main objectives. First, to expand our knowledge of the well-characterized anti-F1 (F1-04-A-G1) and anti-LcrV (7.3) mAbs that have previously been shown to be highly protective in vivo, and to use the antibodies as benchmarks that may later be used to rapidly screen potential novel antibody therapeutics directed against F1 or LcrV antigens. Second, to assess in vitro function and in vivo protective efficacy of a novel anti-plague human polyclonal antibody therapeutic (SAB-183) from genetically engineered cattle, which were vaccinated with the recombinant F1-V subunit vaccine, relative to the anti-F1 (F1-04-A-G1) and anti-LcrV (7.3) mAbs.

Mouse anti-LcrV (7.3) antibody enhanced bacterial uptake by RAW264.7 macrophages. This enhancement was more pronounced when *Y. pestis* was grown at 28 °C for 24 h followed by 37 °C for 2 h (28–37 °C). These growth conditions promote the induction of the T3SS and hence the LcrV antigen production but limit the formation of a robust F1 capsule [18,68]. The level of opsonization of the 28–37 °C grown bacteria after 2 h of invasion was less prominent with anti-F1 (F1-04-A-G1) due to low levels of the F1 capsular protein. The bacteria pretreated with the anti-rF1-V SAB-183 antibodies were phagocytosed to a significantly greater extent than the negative control Abs from unvaccinated Tc bovines (SAB Negative Control, PR1701041NC) (Figure 3). The same trends were observed in the presence of anti-F1 (F1-04-A-G1) mAb pre-incubated with *Y. pestis* grown at 37 °C for 24 h followed by 37 °C for 2 h (37–37 °C), growth conditions that promote a more robust F1 and LcrV production (Figure 4). Since the anti-F1 (F1-04-A-G1) and anti-LcrV (7.3) mAbs are mouse-derived mAbs, it is possible that these mouse IgGs interact with the murine-derived RAW264.7 cells more efficiently than the fully human polyclonal SAB-183 antibodies. Furthermore, since the SAB-183 antibodies are the purified fraction of total IgG from plasma and are not affinity purified against the vaccine antigen, it should come as no surprise that only a fraction of the polyclonal antibodies are directed against the targeted F1 or LcrV antigens. In addition, it is plausible that the chimeric forms of F1 and LcrV in the rF1-V vaccine contain inclusions or exclusions of some of the epitopes that would normally be present in native F1 and LcrV antigens, which may further reduce binding efficacy of the anti-F1 and anti-LcrV fractions of SAB-183. Of note, the LcrV antigen (~326 aa) is more than twice the size of F1 antigen (~150 aa), thereby potentially garnering a greater number of epitopes for immune response after rF1-V vaccination. This size difference in proteins may contribute to the greater antibody titers against LcrV relative to F1 in the SAB-183 antibodies post-vaccination. In addition, antigen availability also needs to be considered since LcrV antigen is naturally expressed at much lower levels relative to F1 antigen. The impact of SAB-183 concentration (100 μg relative to 10 μg) is more pronounced on *Y. pestis* (37–37 °C) relative to *Y. pestis* (28–37 °C). This could be due to overall lower titers against the F1 protein compared to titers directed against LcrV (Table 1). Additionally, the amount of F1 that is produced and released from the bacteria in vivo could result in a decoy effect; thus, requiring a greater concentration of antibody to be effective.

It appears that *Y. pestis* (28–37 °C), due to a lack of a robust capsule along with other temperature-induced factors, is potentially more susceptible to nonspecific antibody binding as seen with much higher invasion in the presence of SAB Negative Control antibodies 2 h post-invasion. However, this enhancement attributed to the SAB negative control is less pronounced with *Y. pestis* (37–37 °C), suggesting the robust F1 capsule formed is obscuring possible cross-reactive antigens on the surface of the bacteria (Figure 3 and Figure 4). Furthermore, in the presence of SAB Negative Control antibodies, this enhancement is also concentration dependent, with an increase in invasion at 100 µg/mL relative to 10 µg/mL for *Y. pestis* (28–37 °C) that is likely attributable to cross-reactive antibodies or possibly endogenous antibodies against other species of *Yersinia*.

Importantly, the human polyclonal antibodies derived from Tc bovines can protect mice from a substantial challenge with aerosolized *Y. pestis*. The studies reported here examined a single administration of antibodies approximately 12 h pre-exposure to aerosolized *Y. pestis*. In future studies it would be important to examine the effect of multiple doses of the antibodies, to determine if these antibodies could be used as postexposure prophylaxis or as a therapeutic, or if the efficacy can be improved with direct delivery to the lungs.

Although a larger amount of this polyclonal antibody material is required to achieve complete protection in the BALB/c mouse model of pneumonic plague (relative to mouse-derived mAbs), even the lower dose (0.5 mg) did significantly increase time-to-death or euthanasia. Whereas mAbs required less material in this mouse model, neither the immunogenicity nor the half-life of mouse-derived mAbs versus fully human antibodies was addressed in this study. Furthermore, the concept of Tc bovines producing antibodies against emerging pathogens is important due to the rapidity of the production and the large amount of material that can be generated. Due to the heterogeneity amongst the LcrV proteins from different isolates [30,75], the level of protection afforded by mAb 7.3 may drastically diminish if that specific epitope is altered; however, greater epitope coverage by polyclonal antibodies derived from Tc bovines may mitigate those possibilities. Additionally, novel antibody-based medical countermeasures will become increasingly more important strategies to combat anti-microbial resistance [76,77,78]. Drug-resistant isolates of *Y. pestis* have been isolated in several regions of Madagascar during relatively recent plague outbreaks and multi-drug resistant isolates continue to be of the utmost concern in both public health and biodefense arenas [79,80]. Thus, novel medical counter measures that limit or prevent *Y. pestis* infection and subsequent plague disease are urgently needed.

## Figures and Tables

**Table 1:** Total IgG antibody response against F1, LcrV, *Y. pestis* CO92 cells or *Y. pestis* C12 cells.

	SAB-183 Neg. Ctrl.		SAB-183 ^a^	
Antigen	Antibody Titer ^b^			
	Median (Q1, Q3)	GEO Mean (GSE)	Median (Q1, Q3)	GEO Mean (GSE)
**F1**	5.0 (5.0, 5.0)	5.0 (1.0)	16,612.6 (16,612.6, 16,612.6)	16,612.6 (1.0)
**LcrV**	10.0 (10.0, 20.0)	12.6 (1.3)	83,063.0 (83,063.0, 83,063.0)	83,063.0 (1.0)
**TS CO92**	320.0 (320.0, 320.0)	320.0 (1.0)	4153.2 (4153.2, 4153.2)	4153.2 (1.0)
**TS C12**	160.0 (160.0, 320.0)	201.6 (1.3)	1038.3 (1308.3, 2076.6)	1308.2 (1.3)

^a^ Antibody titers against all antigens for SAB-183 reached significance (*p* = 0.05) relative to SAB-183 Negative control. ^b^ Values represent median titers with the first and third quartiles (Q1, Q3) and geometric mean (Geo Mean) antibody titer with geometric standard error (GSE) against F1 protein, LcrV protein, *Y. pestis* temperature shifted CO92 (TS CO92) irradiated cells or *Y. pestis* temperature shifted C12 (TS C12) killed cells.

## Data Availability

The raw data supporting the conclusions of this article will be made available by the authors, without undue reservation.

## Supplementary Materials

The following are available online at https://www.mdpi.com/article/10.3390/antib12020033/s1, Figure S1: *Y. pestis* grown overnight at 37 °C has a robust F1 capsule, which is not seen in *Y. pestis* grown for only two hours at 37 °C, Table S1: Statistical analyses for Figure 3 and Figure 4.
