# Supplementary material for: Polyclonal Antibodies Derived from Transchromosomic Bovines Vaccinated with the Recombinant F1-V Vaccine Increase Bacterial Opsonization In Vitro and Protect Mice from Pneumonic Plague
Source: Antibodies (Basel). 2023 May 8;12(2):33. doi: 10.3390/antib12020033 (PMC10204441; doi:10.3390/antib12020033)
Supplement: Supplementary file 1 [file antibodies-12-00033-s001.zip › antibodies-2306077-supplementary.pdf]

Supplemental Figure S1

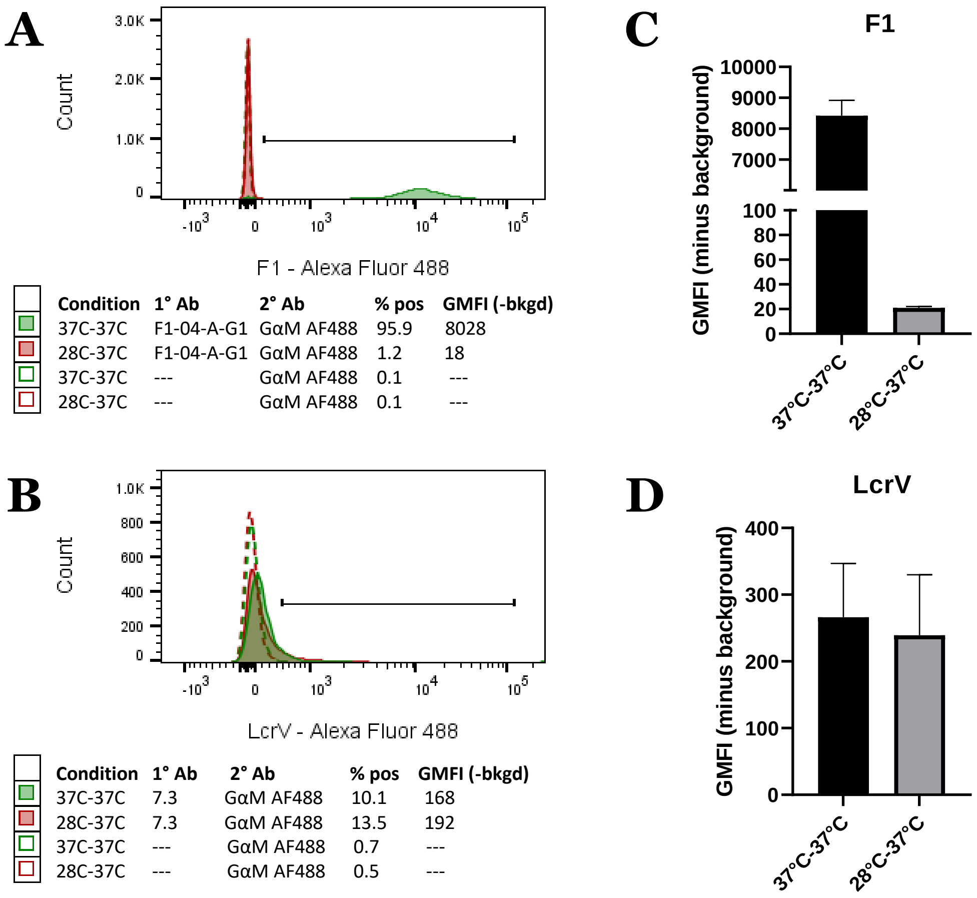

Figure S1. *Y. pestis* grown overnight at 37 °C has a robust F1 capsule, which is not seen in *Y. pestis* grown for only two hours at 37 °C. *Y. pestis* CO92 *pgm-* pPst- (37 °C-37 °C or 28 °C-37 °C) were incubated for 1 h with 10 µg/mL of anti-F1 antibody F1-04-A-G1 or anti-LcrV antibody 7.3, followed by 30 min incubation with goat anti-mouse secondary (Alexa Fluor 488 conjugate). **(A-B)** Bacterial populations were defined based on size and granularity, then quantified for AF488 fluorescence (x-axis). Shown here are representative data for F1 **(A)** and LcrV **(B)** surface staining, comparing F1 and LcrV fluorescence to the background fluorescence of secondary antibody alone. **(C-D)** Staining was quantified as geometric mean fluorescent intensity (GMFI), for four cultures of each growth condition across three independent experiments. After subtracting background, fluorescent intensity measurements were averaged together and plotted for F1 **(C)** and LcrV **(D)**. Bars show the average and SEM of fluorescent intensity for all cultures measured (*n*=4).

Supplemental Table S1: Statistical Analyses for Figures 3 and 4

Y. pestis growth temp.	Expt. Replicate	mAb Conc. (µg)		Yp only	mAb 7.3	mAb F1-04-A-G1	SAB Neg. Ctrl.	SAB-183
28 °C-37 °C	.	10	Wilcoxon p values vs. Yp only		<.0001	<.0001	0.0192	<.0001
		100	Wilcoxon p values vs. Yp only		<.0001	<.0001	0.0001	<.0001
	A	0	n	6				
			Median (Q1, Q3)	24,000 (24,000 , 26,000)				
			GEO Mean (GSE)	24,488.36 (1.06)				
		10	n		6	6	6	6
			Median (Q1, Q3)		150,000 (60,000 , 160,000)	66,000 (40,000 , 200,000)	25,000 (24,000 , 30,000)	69,000 (54,000 , 100,000)
			GEO Mean (GSE)		109,463.22 (1.30)	88,462.18 (1.48)	26,200.42 (1.09)	66,968.26 (1.18)
		100	n		6	6	6	6
			Median (Q1, Q3)		120,000 (80,000 , 180,000)	78,000 (52,000 , 1,000,000)	90,000 (40,000 , 120,000)	140,000 (80,000 , 160,000)
			GEO Mean (GSE)		121,041.93 (1.25)	162,996.97 (1.89)	70,509.38 (1.37)	123,446.55 (1.16)
	B	0	n	6				
			Median (Q1, Q3)	27,000 (24,000 , 30,000)				
			GEO Mean (GSE)	25,350.65 (1.11)				
		10	n		6	6	6	6
			Median (Q1, Q3)		150,000 (140,000 , 200,000)	49,000 (48,000 , 58,000)	60,000 (40,000 , 80,000)	170,000 (120,000 , 200,000)
			GEO Mean (GSE)		154,871.48 (1.12)	50,216.75 (1.06)	58,711.97 (1.19)	157,399.88 (1.13)
		100	n		6	6	6	6
			Median (Q1, Q3)		310,000 (280,000 , 340,000)	90,000 (60,000 , 140,000)	80,000 (60,000 , 140,000)	280,000 (200,000 , 300,000)
			GEO Mean (GSE)		289,188.73 (1.14)	95,711.89 (1.29)	88,603.38 (1.20)	251,657.67 (1.10)
37 °C-37 °C	.	10	Wilcoxon p values vs. Yp only		0.0002	<.0001	0.4845	0.001
		100	Wilcoxon p values vs. Yp only		<.0001	<.0001	0.7011	<.0001
	A	0	n	6				
			Median (Q1, Q3)	4,000 (4,000 , 8,000)				
			GEO Mean (GSE)	4,659.97 (1.27)				
		10	n		6	6	6	6
			Median (Q1, Q3)		8,000 (6,000 , 10,000)	62,000 (36,000 , 100,000)	5,600 (4,000 , 6,000)	18,000 (8,000 , 20,000)
			GEO Mean (GSE)		7,239.81 (1.17)	59,092.28 (1.27)	4,783.52 (1.22)	14,138.36 (1.27)
		100	n		6	6	6	6
			Median (Q1, Q3)		13,000 (10,000 , 18,000)	20,000 (18,000 , 28,000)	4,300 (3,400 , 5,200)	26,000 (20,000 , 26,000)
			GEO Mean (GSE)		12,778.82 (1.14)	22,441.45 (1.15)	4,285.17 (1.11)	23,973.09 (1.08)
	B	0	n	6				
			Median (Q1, Q3)	900 (800 , 1,000)				
			GEO Mean (GSE)	323.77 (3.20)				
		10	n		6	6	6	6
			Median (Q1, Q3)		11,000 (8,000 , 16,000)	43,000 (42,000 , 50,000)	1,600 (1,400 , 2,000)	6,200 (4,800 , 6,800)
			GEO Mean (GSE)		10,880.17 (1.19)	43,721.89 (1.05)	1,431.41 (1.20)	5,748.82 (1.12)
		100	n		6	6	6	6
			Median (Q1, Q3)		24,000 (22,000 , 24,000)	35,000 (32,000 , 56,000)	1,300 (1,000 , 1,600)	44,000 (40,000 , 48,000)
			GEO Mean (GSE)		23,254.66 (1.04)	41,107.75 (1.24)	1,275.24 (1.09)	43,693.69 (1.05)
